# Cell Cycle Regulation in Macrophages and Susceptibility to HIV-1

**DOI:** 10.3390/v12080839

**Published:** 2020-07-31

**Authors:** Isabella A. T. M. Ferreira, J. Zachary Porterfield, Ravindra K. Gupta, Petra Mlcochova

**Affiliations:** 1 Cambridge Institute of Therapeutic Immunology & Infectious Disease (CITIID), Cambridge CB20AW, UK; iatmf2@cam.ac.uk (I.A.T.M.F.); rkg20@cam.ac.uk (R.K.G.); 2 Department of Medicine, University of Cambridge, Cambridge CB20QQ, UK; 3 Department of Microbiology, University of Kentucky, Lexington, KY 40536, USA; zach.porterfield@uky.edu; 4 Africa Health Research Institute, Durban 4001, South Africa

**Keywords:** HIV, SAMHD1, macrophage, cell cycle, cell arrest, G0/G1 phase

## Abstract

Macrophages are the first line of defence against invading pathogens. They play a crucial role in immunity but also in regeneration and homeostasis. Their remarkable plasticity in their phenotypes and function provides them with the ability to quickly respond to environmental changes and infection. Recent work shows that macrophages undergo cell cycle transition from a G0/terminally differentiated state to a G1 state. This G0-to-G1 transition presents a window of opportunity for HIV-1 infection. Macrophages are an important target for HIV-1 but express high levels of the deoxynucleotide-triphosphate hydrolase SAMHD1, which restricts viral DNA synthesis by decreasing levels of dNTPs. While the G0 state is non-permissive to HIV-1 infection, a G1 state is very permissive to HIV-1 infection. This is because macrophages in a G1 state switch off the antiviral restriction factor SAMHD1 by phosphorylation, thereby allowing productive HIV-1 infection. Here, we explore the macrophage cell cycle and the interplay between its regulation and permissivity to HIV-1 infection.

## 1. Introduction

HIV-1 infection is suppressed but not cured in the face of antiretroviral therapy (ART) due to sanctuary sites throughout the body termed the HIV reservoir [1,2,3]. Within these sites, different cell types are responsible for maintaining infection through different mechanisms such as ongoing replication or latency [4,5,6]. CD4 T cells are most commonly referred to as the cellular HIV reservoir; however, there are other cell types that are also involved, including macrophages [7,8,9,10]. Despite three decades of intense HIV-1 research, only two patients have been cured, requiring allogeneic stem cell transplantation and associated chemotherapy [11,12,13]. This likely reflects the complexity of the mechanisms at play in the persistence the HIV reservoir.

Macrophages are of paramount importance in defending the host against viruses and other pathogens, in addition to having roles in homeostasis, tissue repair and development [14,15]. Furthermore, they are found in multiple tissues throughout the body, including anatomical sanctuary sites such as the brain [16,17], a site with lower drug penetrance [18,19]. As a result, HIV-1 is able to persist in these anatomical sanctuary sites, even in presence of ART [20,21]. Furthermore, HIV-1 is less cytopathic to macrophages than to CD4 T cells. It has been suggested that the HIV infection of MDM (monocyte-derived macrophages) does not activate apoptotic effector caspases [22] or downregulates the death ligand TRAIL (TNF-related apoptosis inducing ligand) [23]. Due to the long half-lives of macrophages, HIV is able to persist in these cells and rebound from these cells when ART is interrupted [8,15].

In this review, we will address recent research showing that macrophages, normally an unfavourable environment for HIV-1 infection, can become permissive to HIV-1 infection through naturally occurring modulations in their cell cycle. Furthermore, we discuss new strategies targeting the host cell cycle that could be manipulated to achieve a non-permissive state, block HIV-1 infection and thus potentially limit the macrophage cellular reservoir.

## 2. Macrophage Origin and Polarization/Activation

Macrophages are professional phagocytic cells that play a role in a wide range of biological processes. They possess a variety of functions including roles in immunity and inflammation but also in tissue homeostasis and regeneration [24].

### 2.1. Origin

Historically, macrophages were considered to originate from hematopoietic stem cells via bone marrow progenitors and circulating monocytes. It was believed that adult tissue-resident macrophages originated from and were replenished by circulating monocytes. In contrast with this long-held view, it has become evident that during embryogenesis, macrophages colonise developing organs, and as they have a capacity to self-renew, they remain there even in adult tissue [25,26,27,28]. Thus, there is a dual origin of macrophages, both from embryonic progenitors and from circulating blood monocytes.

Despite these data, most studies on macrophage origins have been undertaken in mouse models. The exact origin of macrophages in humans remains to be elucidated. The study of human macrophage ontogeny faces several limitations since we cannot use fate-mapping and do not have good markers to discriminate between tissue-resident and monocyte-derived macrophages that have entered the tissue in question, making it impossible to characterize the macrophage origin in humans. Nevertheless, recent transplantation studies have provided some new views and have shown that donor-derived macrophages persist for many years in transplanted skin or liver in patients [29]. Furthermore, lung macrophages can self-renew for years in a murine model [30,31], but recent work on alveolar macrophage ontogeny in the human lung during healthy aging and after transplant suggests that donor alveolar macrophages are quickly replaced from the recipient peripheral circulating monocytes [32]. Interestingly, the human myocardium contains two distinct subsets of macrophages: CCR2− and CCR2+ (C-C chemokine receptor type 2). While CCR2− macrophages are tissue resident and can self-renew, CCR2+ macrophages are maintained from circulating monocytes through localized cell proliferation [33].

### 2.2. Localization

The tissue localization of macrophages is a complex area; macrophages have different designations in different tissues such as microglial cells (brain), Langerhans cells (skin) or alveolar macrophages (lung), but diverse tissue microenvironments can shape the active states and polarization of macrophages and influence their function and phenotypes. The two most commonly reported phenotypes are M1 and M2 macrophages, otherwise known as pro-inflammatory and anti-inflammatory or classically and alternatively activated macrophages. Nevertheless, diverse microenvironments affect the polarization of macrophages in a way that renders M1 and M2 nomenclature obsolete, and diverse populations of macrophages ranging between the two extremes of M1 and M2 are frequently reported. Furthermore, it has been reported that the M1 and M2 activation of macrophages in vitro does not match polarization in vivo [34].

Moreover, current available data provide a picture where macrophages possess high phenotypic heterogeneity and functional plasticity that is mediated by microenvironmental signals but could also be triggered by surrounding cells [35,36,37].

A recent study compared gene expression between ex vivo and in vitro human microglia. It has been shown that the transfer of human microglia into an in vitro environment results in the profound alteration of the expression of thousands of genes [35], supporting the idea that macrophages can quickly adapt to a new environment due to epigenetic modifications.

Furthermore, emerging evidence shows that tumour-associated macrophages (TAMs) can acquire a whole spectrum of functional, metabolic and phenotypic profiles in response to environmental changes inside tumours [37,38,39]. Lavin et al. showed that TAMs in early lung adenocarcinoma display a distinct transcriptional signature compared to their lung-resident counterparts and thus provided insights into how the tumour microenvironment reshapes human lung macrophages [36].

Remarkable macrophage plasticity allows them to respond to environmental signals and alter their functions, phenotype and activation. It can provide them with enhanced antimicrobial activity or increased immune functions, but it can also increase susceptibility to pathogenic infection [40].

## 3. Macrophages and HIV Infection

Most published macrophage research uses in vitro murine model bone-marrow-derived macrophages or human monocyte-derived macrophages (MDM). Even though MDM might not be the most accurate model for tissue-resident macrophages, they are still a well-accepted model for human primary macrophages for studying immunity and infection, e.g., in HIV infection.

Several important studies show that macrophages are permissive to HIV-1 infection and can sustain infection even in virologically suppressed individuals [9,41,42,43,44,45,46,47]. Macrophages form a stable long-lived reservoir in tissues such as the urethra, vaginal tract and gut, allowing HIV-1 to persist [8,9,48,49].

A recent example of the macrophage HIV-1 reservoir was elucidated in urethral macrophages. Ganor et al. (2019) showed that HIV RNA is present in a subset of macrophages in the urethra, detecting *Nef* and *Alu* repeats using FISH (fluorescence in situ hybridisation) as well as integrated HIV DNA using *Alu-gag* PCR. Furthermore, in this study and other studies, HIV-1 virions have been detected in macrophages inside intracellular virus-containing compartments, such as intracellular vacuoles, suggestive of productive infection [9,10]. The quantitative viral outgrowth assay showed that HIV was reactivated from urethral macrophages by lipopolysaccharide (LPS). However, PHA (phytohemagglutinin) could not reactivate any HIV-1 from CD3+ cells from the urethral tissue site, indicating that urethral T cells do not form the HIV reservoir at this site. Whilst the urethra was found to have extreme polarisation of M1 and M2 macrophages, many unique intermediate macrophage subsets were found to contain HIV-1, confirming the tissue plasticity of this macrophage reservoir [9].

Macrophages present a hostile environment for viral infection. They express highly active immune defences, including HIV restriction factors such as a SAM domain and HD domain-containing protein 1 (SAMHD1). SAMHD1 is a deoxynucleotide triphosphohydrolase [50] that plays a role in DNA metabolism [51] as well as in DNA repair processes [52]. A widely accepted mechanism of HIV restriction by SAMHD1 is the depletion of dNTPs to levels that are insufficient to allow viral DNA synthesis [50,53,54,55]. The hydrolase activity of SAMHD1 was shown to be negatively regulated by CDK1-, CDK2-, CDK4- and CDK6-mediated phosphorylation at amino acid T592 [56,57,58], and positively regulated by the PP2A-B55a-mediated dephosphorylation of SAMHD1 [59].

Some lentiviruses have evolved countermeasures against SAMHD1; for example, the HIV-2 and SIVsm lineage encodes the Vpx protein, which degrades SAMHD1 and allows the infection of otherwise SAMHD1-positive target cells [60,61,62]. How pandemic HIV-1 strains achieve the efficient infection of terminally differentiated macrophages in vivo where SAMHD1 is active without a Vpx-like activity has remained a significant unresolved question that has limited our understanding of HIV tropism and pathogenesis [57,63].

## 4. Cell Cycle Regulation in Macrophages

### 4.1. G0-to-G1 Transition: A Window of Opportunity for HIV-1 Infection

Our previous work investigating viral infection in primary human macrophages has revealed that macrophages undergo cell cycle transition from a G0/terminally differentiated state to a G1 state, without actually dividing [57]. A comparison of transcriptional profiles for a predefined gene signature that discriminates macrophages from other cell types [64] showed that G0 and G1 macrophages cluster together and are distinct from closely related myeloid cells [57].

**Figure 1:**
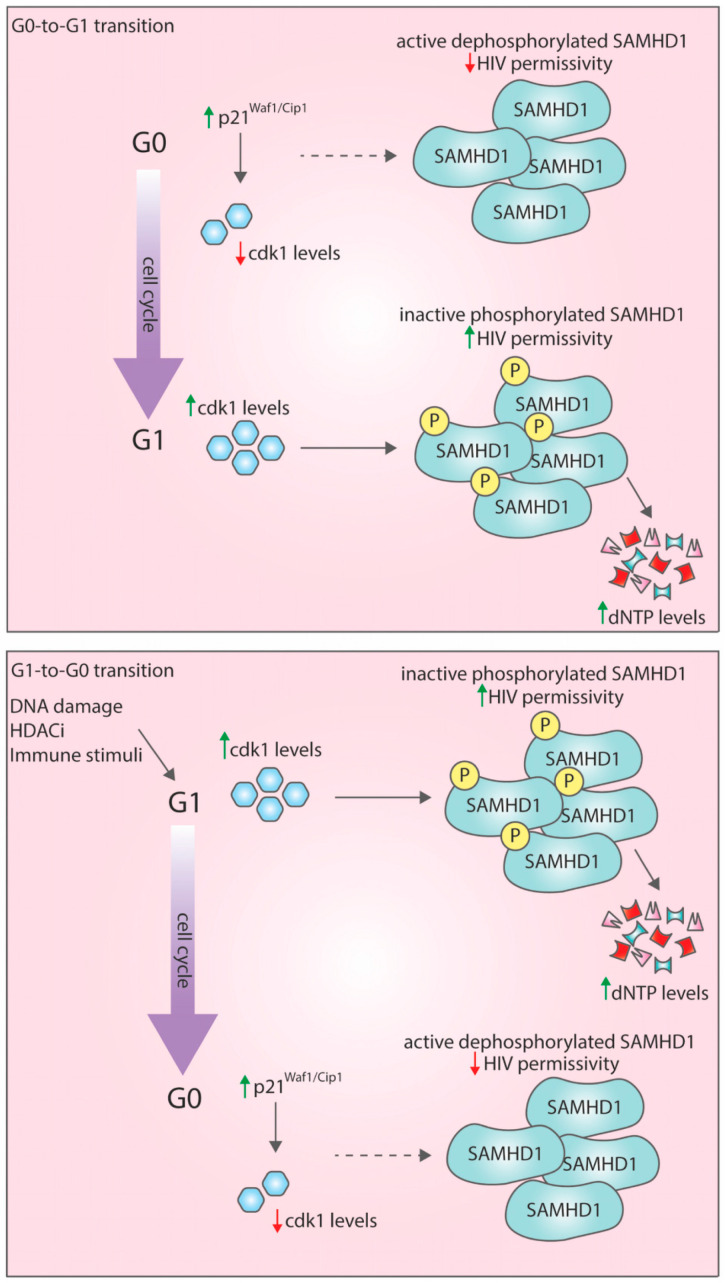
G0-to-G1 transition presents a window of opportunity for HIV-1 infection. G0-to-G1 transition: Macrophages in the G0 state express the negative cell cycle regulator p21(Waf1/Cip1). In this state, CDK1 levels are low and SAMHD1 is activated/dephosphorylated, thus decreasing dNTP levels. Macrophages in this state are highly refractory to HIV-1 infection. The activation of the MEK/ERK signalling pathway triggers monocyte-derived macrophage (MDM) entry to a G1 state where p21 is downregulated and CDK1 is expressed and inactivates SAMHD1 by phosphorylation. The dNTP levels are increasing, and the macrophages are permissive to HIV-1 infection. G1-to-G0 transition: When MDM in G1 state are exposed to danger signals (e.g., DNA damage, HDACi and immune stimuli), they can revert back to the G0 state. This is accompanied by an increase in p21 levels, CDK1 downregulation and SAMHD1 dephosphorylation/activation, leading to the blocking of HIV-1 infection.

Intriguingly, G0-to-G1 transition presents a window of opportunity for HIV-1 infection. We have shown that the G0 state is non-permissive to HIV-1 infection, but the G1 state is very permissive to HIV-1 infection [57]. We have discovered that this is because macrophages in a G1 state switch off the antiviral restriction factor SAMHD1 by phosphorylation, thereby allowing productive HIV-1 infection (Figure 1). This answered the long-standing question of how HIV-1 could infect macrophages even though they express high levels of this restriction factor [56,57,65].

**Figure 2:**
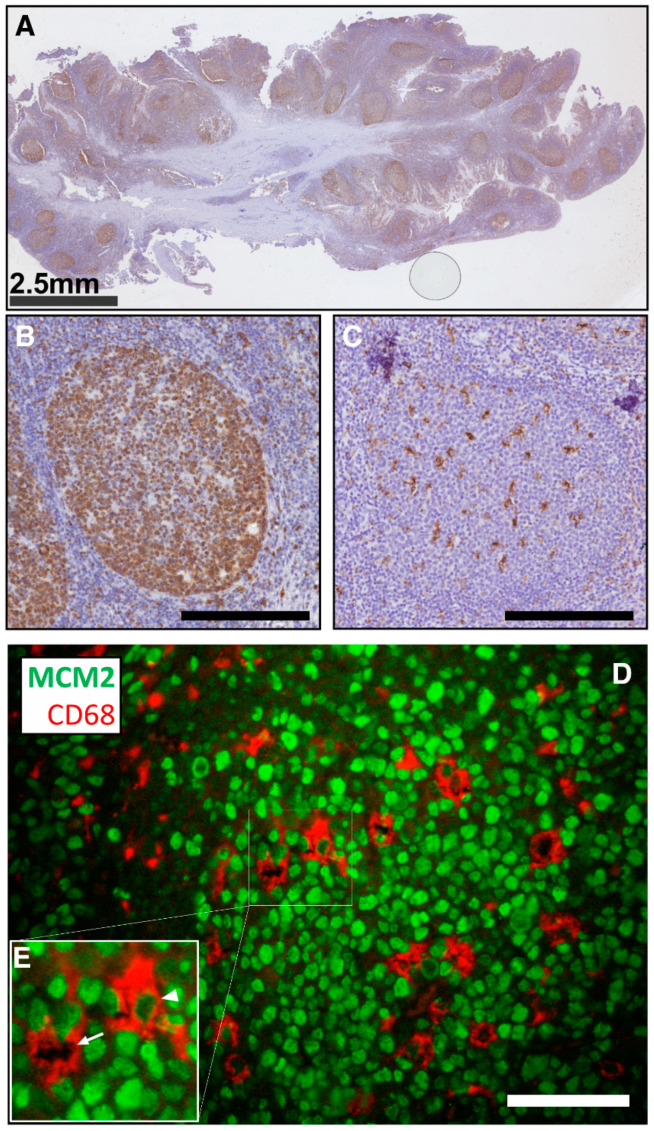
MCM2-expressing macrophages in human tonsils. (**A**–**C**) Immunohistochemistry of tonsil sections. (**A**) Low magnification of full tonsil section stained for MCM2, highlighting germinal centres. (**B**) Higher magnification of a germinal centre strongly stained for MCM2. Scale bar: 300 μm. (**C**) A germinal centre stained for CD68 (macrophage marker), highlighting germinal centre macrophages. Scale bar: 300 μm. (**D**,**E**) Immunofluorescence staining of a tonsil germinal centre. MCM2 (green), a marker of cell cycle entry and proliferation. Macrophages are indicated by positive CD68 staining (red). Macrophages are observed as both positive (arrowhead) and negative (arrow) for MCM2 (a nuclear protein). Scale bar: 50 μm. (**E**) High-magnification image of boxed region.

Furthermore, we have shown that murine macrophages isolated from the brain or peritoneum also exist in G0 and G1 states in vivo [57]. Critically, we have identified MCM2 (mini-chromosome maintenance protein 2)-positive macrophages in human lymph nodes, supporting the role of G0-to-G1 transition in a human model and tissue-resident macrophages (Figure 2).

Furthermore, we have established a tractable model whereby the transition between these states can be easily manipulated in human monocyte-derived macrophages in vitro, allowing us to study processes that might have an impact not only in infection but also in fundamental macrophage biology [57].

### 4.2. G1-to-G0 Transition

Cells have many different mechanisms by which they protect themselves from incoming danger, including chemical, physical or biological threats (e.g., infections). In view of our recent discoveries [66] that macrophage G1-to-G0 transition occurs following DNA damage, HDAC inhibition (histone deacetylation inhibition) and immune stimuli, we speculate that cell cycle regulation might be a conserved and principal cell defence response to danger signals in human monocyte-derived macrophages.

### 4.3. Histone Deacetylase Inhibitors (HDACi)

HDACi are a class of compounds that inhibit histone deacetylases and play a major role in the transcriptional regulation of cells by altering the acetylation status of histone and non-histone proteins. HDAC inhibitors also induce cancer cell arrest, differentiation and cell death and reduce angiogenesis, which makes them important molecules in cancer treatments; as such, HDACi have been used in clinical trials against a variety of cancers [67,68,69]. In addition to their known effects on gene transcription [70], they can also reactivate HIV from latent reservoirs [71,72,73].

As we aim to achieve sustained HIV remission, it is desirable to induce a state of cellular resistance to HIV infection by host-directed therapy in addition to antiviral therapy. We showed that in addition to HDACi’s known effects on gene transcription and HIV reactivation through histone modification, these inhibitors activate SAMHD1 antiviral activity through the regulation of the cell cycle in macrophages.

We have reported that HDACi treatment in macrophages resulted in the upregulation of p53 and p27, which correlated with the loss of MCM2 and CDK1 expression as well as SAMHD1 dephosphorylation, suggestive of G1-to-G0 transition, with no observed cytotoxicity [74]. HDACi also inhibited HIV-1 infection in macrophages in a SAMHD1-dependent manner.

The ability of HDACi to reactivate latent virus and, at the same time, to prevent the infection of new target cells is a major advantage of these agents as a means of achieving a functional HIV cure.

### 4.4. DNA Damage

Recently, it has been shown that DNA damage (mediated by topoisomerase inhibitors used in cancer treatment—e.g., etoposide—or by UV light) is connected to the SAMHD1-dependent inhibition of HIV-1 infection in primary human monocyte-derived macrophages [57,75]. It has been demonstrated that DNA damage activated p21 in macrophages [57,75], leading to the inhibition of CDK1/2 and SAMHD1 dephosphorylation. SAMHD1 in this state was able to restrict HIV-1 infection. As expected from our previous data, this loss of CDK1 activity and SAMHD1 phosphorylation was mediated by the transition of G1 macrophages back to a G0 state. We also demonstrated in this work that macrophage infection by Vpx-encoding viruses such as SIV was not impacted by DNA damage agents. Importantly, G1-to-G0 transition was not accompanied by etoposide-induced cell death or by apoptosis.

Chemotherapeutic agents such as etoposide are used in HIV-infected individuals for malignancies such as lymphoma [76]. It would be informative to explore if and how DNA damage responses could shield myeloid cell populations from HIV infection. Such new insights might aid in the design of novel therapeutic interventions, especially for central nervous system reservoirs that are mostly populated by macrophages and related myeloid lineages [77,78].

### 4.5. Immune Stimuli and Gram-Negative Bacteria

Lipopolysaccharide, which is found on the outer membrane of Gram-negative bacteria, is known to block macrophage proliferation by inducing cell cycle arrest in mouse primary cells and murine cell lines [79,80] or in the human cell line THP-1/U937 [81,82,83,84]. Our most recent work showed that primary human macrophages respond to gram-negative bacteria and LPS (lipopolysaccharide) not only by activating a TLR4 pathway culminating in NFkB and IRF3 activation but also by a novel pathway culminating in p21 upregulation and G1-to-G0 transition. This previously unrecognised pathway is interferon-independent. These data suggest that macrophages can rapidly reach a state of alert in response to Gram-negative bacteria that is triggered prior to type I interferon secretion [66].

Even though we have previously reported that MDM re-enter the cell cycle into the G1 phase without measurable cell division, many tissue-resident macrophages have the ability to proliferate [85]. We could speculate that the division of a cell harbouring live pathogens would lead to the multiplication of infected cells, an event potentially detrimental to the cell/host. Hence, cell arrest could be a defence mechanism for limiting the local invasion of Gram-negative bacteria in macrophages.

We showed that TLR-4 pathway activation in MDM and G1-to-G0 transition was accompanied by the dephosphorylation/activation of SAMHD1 and blocking of HIV-1 infection in macrophages. Macrophages such as those in the gut are an important cellular reservoir of HIV [86]. It is possible that macrophages may become non-permissive when exposed to gut-derived LPS during inflammation in both the acute and chronic phases of HIV infection. This interferon-independent regulation of SAMHD1 by TLR4 activation represents a novel mechanism for reducing the HIV-1 reservoir size and may contribute to curative interventions.

While G0-to-G1 transition presents a permissivity window for HIV to infect macrophages, it is evident that this window can be closed when macrophages face danger. Investigating and understanding these processes would allow us to design new strategies where host cells can be re-programmed to become non-permissive to infection, thus limiting cellular reservoirs, even in sanctuary sites such as the brain [87,88].

## 5. HIV-1 Regulates the Host Cell Cycle

G0-to-G1 transition seems to be a fundamental biological function of MDM that is independent of viral infection. Many viruses have the ability to subvert the cell cycle directly by themselves [89,90,91], and HIV is no exception to this. The HIV-1 accessory proteins Vpr and Vif have been shown to alter the cell cycle in cycling cells to prime the host cell capacity to support viral infection.

### 5.1. Viral Protein R (Vpr)

Vpr is an accessory protein present in HIV-1, HIV-2 and SIVs. Even though the evidence for Vpr’s importance in efficient viral replication, especially in primary human macrophages, is inconclusive [92,93,94], many studies have revealed important new roles for this accessory protein in HIV pathogenesis. Vpr is best known for inducing DNA damage responses and G2-M cell cycle arrest in cycling cells. Vpr-induced cell cycle arrest at the G2 phase can provide an in vivo replicative advantage for HIV-1 as evidence suggests that viral genome expression is optimal in the G2 phase of the cell cycle [95,96,97].

Several recent studies have investigated different cellular mechanisms and pathways whereby Vpr is able to block the G2-to-M transition and subsequently arrest the cell cycle. Firstly, Vpr was suggested to arrest the cell cycle by exploiting and activating the ATR signalling pathway and ATR-dependent G2 checkpoint. Vpr-mediated G2 arrest specifically requires Hus1 and Rad17 to induce G2 arrest along with the phosphorylation of H2AX [98]. Secondly, Laguette et al. (2014) proposed a mechanism where Vpr induces G2/M cell arrest through the activation of the structure-specific endonuclease regulator SLX4 complex, which is involved in DNA repair [99]. Vpr’s direct interaction with SLX4 recruits VprBP/DCAF1 (a component of E3 ubiquitin-protein ligase complexes) and pLK1 to augment MUS81–EME1 endonuclease function [99]. MUS81 and EME1 play a role in rescuing stalled replication forks, and their premature activation can cause replication stress and lead to cell cycle arrest [99,100].

Lastly, several studies have shown that Vpr triggers G2 arrest by hijacking the Cul4/DDB1^DCAF1^ E3 ubiquitin ligase [99,101,102,103,104,105,106] and revealed new targets for Vpr-mediated degradation [101,106]. Among these newly identified Vpr targets are proteins closely connected to DNA repair such as the HLTF DNA helicase and the multifunctional Exo1 nuclease [106]. Additionally, the proteins SMN1 and CDCA2 are known to activate the ATM/ATR-dependent DNA damage response pathway [101]. These data support the link between DNA damage and Vpr-mediated cell cycle arrest. Interestingly, Greenwood et al. (2019) showed that Vpr degrades multiple targets, causing changes to the cellular proteome and, as a consequence, to many cellular pathways instead of being restricted to only G2 cell cycle arrest. This promiscuous targeting of multiple host factors underpins the complex Vpr-dependent cellular phenotype and might explain why Vpr’s effects on cellular phenotypes and viral replication remain inconsistent.

### 5.2. Viral Infectivity Factor, Vif

Vif is an accessory protein best known for targeting APOBEC3G/F for ubiquitination and degradation to prevent its antiviral activity and thereby increasing the infectivity of viral particles [107,108,109]. Nevertheless, Vif has also been reported to delay the G2 phase of the cell cycle [110] or cause G2 cell cycle arrest, even in the absence of Vpr [109,110]. This G2 cell cycle arrest does not require the expression of APOBEC3, but it is dependent on Cullin 3 ubiquitin ligase engagement and proteasome function [107,109,110,111,112]. Importantly, Vif-triggered cell cycle arrest seems to create an environment favourable for HIV replication [113].

Izumi et al. showed that Vif interacts with the MDM2/TP53 pathway, a pathway that is involved in regulating the G2/M transition in response to genotoxic stress. Vif enhances the stability of TP53 as well as its transcriptional activity by preventing the MDM2-mediated ubiquitination and nuclear export of TP53 to induce cell cycle arrest [107]. Furthermore, Sakai et al. showed that the dephosphorylation of an inhibitory phosphate on CDK1 did not occur in infected cells expressing Vif and that CDK1-CyclinB1 nuclear translocation was impaired. As CDK1-CyclinB1 proteins are essential for initiating mitotic entry, Vif thus arrests cells in the G2/M phase [111].

The latest research indicates that Vif has the ability to remodel the phosphoproteome of HIV-infected cells [114]. Vif was found to recruit the same Cul5 E3 ubiquitin ligase complex that it uses to target APOBEC3C to degrade PP2A phospho-regulators (PPP2R5), which modulates the cell cycle. PP2A counteracts mitotic kinases that are essential to secure the progression of the cell cycle, including CDK1, aurora and PKL1 [115,116,117,118]. Indeed the Vif-dependent depletion of PPP2R5A causes an increase in protein phosphorylation in HIV-infected cells, including that of substrates of the aurora kinases and CDK1 [114]. It has been concluded that the depletion of the PPP2R5 family subunits is necessary for Vif-dependent cell cycle arrest [101,113,119,120].

### 5.3. What Is the Role of Vpr/Vif-Mediated G2/M Arrest in Terminally Differentiated Cells Such as Macrophages?

Vpr/Vif-mediated G2/M arrest studies have been performed in cycling cells including T cell lines [107,109,111,113,121,122], THP-1 [99], Hela and HEK293 [98,113,122], and primary CD4 T cells [120,123].

What about terminally differentiated cells such as macrophages? Can HIV-1-mediated G2/M arrest affect MDM? As terminally differentiated cells do not progress to G2/M or proliferate, it would be highly improbable that G2/M arrest by viral accessory proteins would play any important role. Nevertheless, recent studies show that tissue-resident macrophages are not terminally differentiated. Tissue-resident macrophages have the ability to self-renew and progress to and through the G2/M phase. In these cells, Vpr/Vif could play an important role in cell cycle arrest and priming the host cell for optimal infection and replication. As we still have limited knowledge of HIV infection in tissue-resident macrophages, further research including cell cycle progression and arrest studies, including the role of G1-to-G0 transition after danger stimuli—e.g., DNA damage, HDAC inhibition and immune stimuli—will be of high importance for our understanding of HIV reservoirs in macrophages.

## 6. Conclusions

Macrophages have been shown to contribute to the HIV reservoir in sanctuary sites such as the brain and gut. This is due to their longevity and their ability to resist the cytopathic effects of HIV. Even though macrophages are typically portrayed as cells resistant to HIV infection due to low dNTP levels and the restriction factor SAMHD1, others and we have shown that macrophages can regulate dNTP levels and allow productive HIV-1 infection.

In vitro and in vivo studies show that macrophages are able to transition from a G0 to a G1 state, regulate SAMHD1 activity and render macrophages highly permissive to HIV infection. As G0-to-G1 transition present a window of opportunity for HIV infection, could we manipulate this process to prevent cells becoming infected? There have been reported several mechanisms whereby macrophages are able to arrest the cell cycle and/or revert from a G1 back to a G0 state and block HIV-1 infection. These includes treatment with HDAC inhibitors, exposure to DNA damage or immune stimuli. We propose that G1-to-G0 transition is a defence mechanism of the cell to protect itself from danger, including invading pathogens.

Eradicating the HIV-1 reservoir is seen as a tractable means for achieving long-term remission/cure. The role of macrophages has been relatively understudied, and we believe that therapeutic advances may be made by furthering our understanding of the mechanisms of HIV infection in tissue-resident macrophages, particularly in relation to cell cycle regulation.
